# Neutrophil-to-ferritin ratio can predict hematological causes of fever of unknown origin

**DOI:** 10.1038/s41598-024-74569-0

**Published:** 2024-10-03

**Authors:** Hikmet Öztop, Fazıl Çağrı Hunutlu

**Affiliations:** 1 Department of Internal Medicine, Faculty of Medicine, Bursa Uludag University, Gorukle Campus, Bursa, Turkey; 2 Division of Hematology, Department of Internal Medicine, Faculty of Medicine, Bursa Uludag University, Bursa, Turkey

**Keywords:** FUO, Lymphoma, Ferritin, Inflammation, Diagnostic markers, Predictive markers, Lymphoma

## Abstract

Despite advancements in diagnostic modalities, delineating the etiology of fever of unknown origin (FUO) remains a significant challenge for clinicians. Notably, cases with hematological malignancies often have a poor prognosis due to delayed diagnosis. This study investigated the potential of readily obtainable laboratory markers to differentiate hematological causes from other etiologies during the early stages of FUO. A retrospective analysis was conducted on the medical records of 100 patients who fulfilled the modified FUO criteria between January 2010 and April 2023. Hematological etiologies were identified in 26 of the 100 patients. Peripheral blood neutrophil, lymphocyte, platelet counts, and the systemic immune inflammation (SII) index, were significantly lower in the hematological group compared to the non-hematological group. Conversely, serum ferritin levels were demonstrably higher in the hematological group. ROC analysis identified a neutrophil-to-ferritin ratio (NFR) cutoff value of < 8.53 as optimal for predicting hematological etiology. Subsequent multivariate analysis demonstrated that the NFR was the sole independent predictor of hematological etiology (*p* = 0.013).This study proposes a novel approach for early diagnosis of a potentially life-threatening subset of FUO patients. The NFR presents as an inexpensive and readily available marker for predicting hematological etiology in FUO cases.

## Introduction

The diagnostic criteria for fever of unknown origin (FUO) were first defined by Petersdorf and Beeson in 1961 and revised by Durak and Street in 1991^1^. According to the latest revised diagnostic criteria, FUO is defined as a fever of 38.3 °C or higher that at least 3 week or that cannot be diagnosed after an inpatient hospitalization for 3 days or three outpatient follow-up visits. In addition to classic FUO cases, nosocomial, neutropenic, and HIV-related FUO cases were also defined in the same revision^2^. The differential diagnosis of FUO spans over 200 potential pathologies^3^. The main etiologic groups include infections, malignancies, non-infectious inflammatory diseases, and other causes for undiagnosed patients despite all necessary investigations^4–6^. Global epidemiological trends reveal variations in FUO etiologies, with infections and malignancies more prevalent in low-income countries and non-infectious inflammatory diseases and undiagnosed cases predominant in high-income settings^3,4,6^.

An individualized approach is always recommended for diagnosing and treating FUO cases^7^. Despite advancements in diagnostic technologies, a notable proportion of FUO cases remain undiagnosed, particularly those attributed to malignant etiologies, which carry a poor prognosis^8–10^. Among FUO patients, approximately 10–15% are diagnosed with hematologic malignancies, characterized by high mortality rates^10^. Bone marrow biopsy, a crucial diagnostic tool, yields a diagnostic value of around 25% in these cases^11,12^. Given the invasiveness of such procedures, there is a growing need for non-invasive diagnostic markers to predict hematologic malignancies.

Despite individualized diagnostic approaches and sophisticated tests, establishing the precise etiology of FUO remains challenging, leading to prolonged diagnostic timelines and increased mortality rates, especially in cases involving malignancies. Efforts to streamline diagnostic processes and enhance cost-effectiveness underscore the necessity for accessible markers that could offer insights into underlying etiologies. We aimed to investigate the potential clinical and laboratory parameters to predict hematologic etiologies among hospitalized FUO patients.

## Materials and methods

The study included patients over 18 years of age who were evaluated for FUO by the Internal Medicine Clinic of Bursa Uludag University Faculty of Medicine between January 2010 and April 2023. Only patients who met the modified FUO criteria were included in the study. The study comprises classical FUO according to the FUO criteria, excluding nosocomial, neutropenic, and HIV-related FUO cases^2^. Demographic data, comorbidities, clinicopathologic features, laboratory parameters, imaging, and pathology results at hospitalization were obtained retrospectively from patient files and the hospital data processing system.

**Fig. 1:**
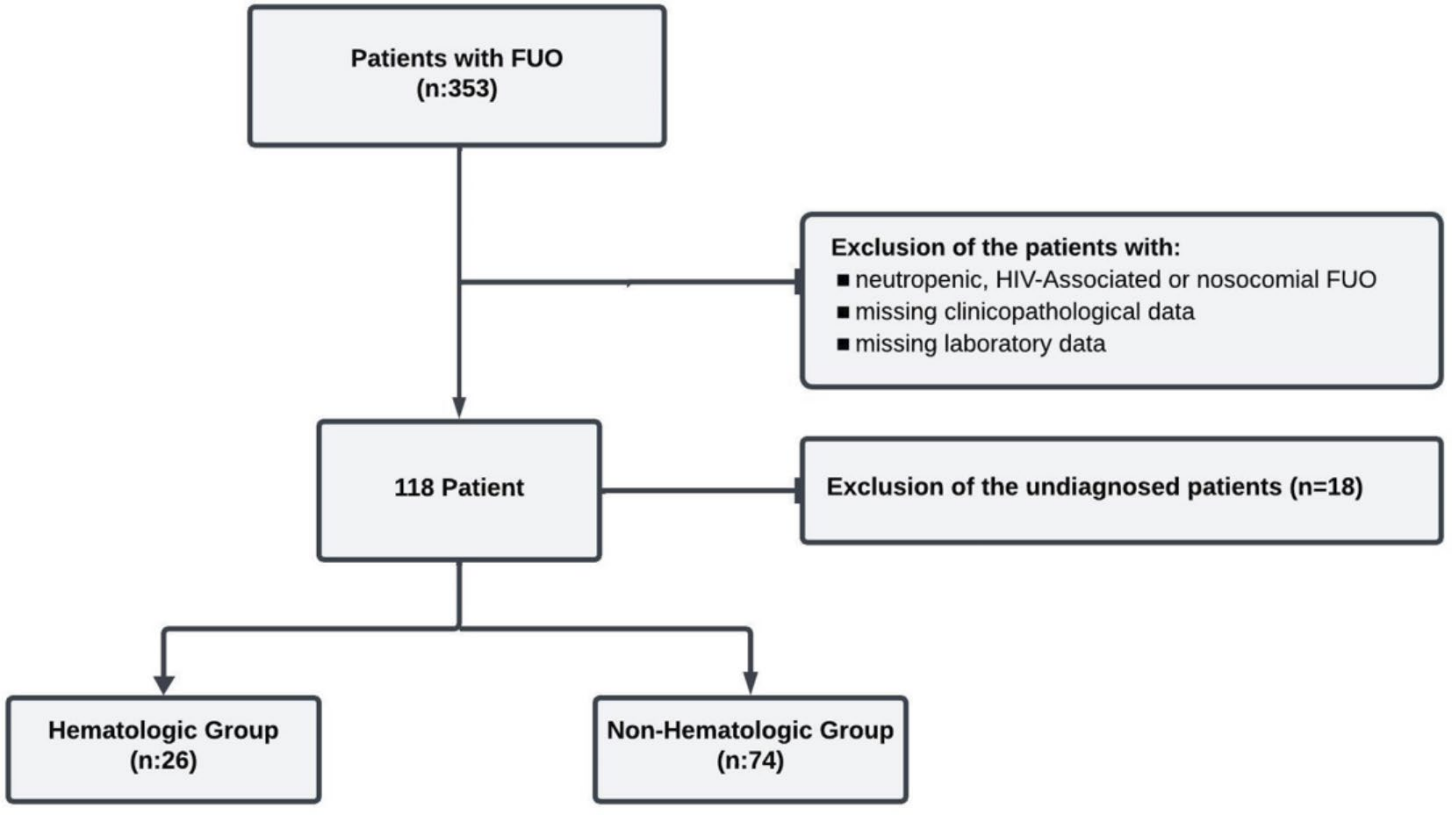
Flow-chart of the study.

Figure 1 shows the flow chart of the patients. Standard diagnostic procedures were performed in all patients, including blood and urine culture for infectious agents, microbiologic serologic tests, ultrasonography, or computed tomography as an imaging modality, as well as PET-CT, bone marrow, and lymph node/tissue biopsy if indicated. The diagnosis of infection was confirmed by bacterial culture, serologic/molecular tests, radiology, or histopathology. Hematologic or solid organ malignancy was diagnosed by tissue biopsy. Rheumatologic diseases and other rare causes were diagnosed using classification criteria, including clinical findings, biochemical tests, autoantibody panels, and imaging modalities^13–16^. Possible concomitant infectious agents were excluded from the non-infectious group after microbiologic and radiologic examinations. Patients who were unable to be diagnosed after minimal and advanced FUO evaluation^17,18^, or who died during the FUO diagnostic examinations, were categorized as undiagnosed. In our study, which aimed to predict hematologic etiology, 18 patients in the undiagnosed group were excluded from the study to prevent potential bias in the statistical analysis process. Neuthrophil-lymphocyte ratio (NLR) was calculated as neutrophil count/lymphocyte count, platelet-lymphocyte ratio (PLR) as platelet count/lymphocyte count, the systemic inflammatory index (SII) as platelet count × neutrophil count/lymphocyte count, and the systemic inflammation response index (SIRI) as neutrophil count × monocyte count/lymphocyte count in peripheral blood. Neutrophil-to-ferritin ratio (NFR) was calculated by dividing neutrophil count by ferritin. The records of the patients in the nonhematologic group were examined prospectively after the diagnosis of FUO, confirming that malignancy was not observed in at least 1 year.

### Statistical analysis

Data were analyzed using SPSS version 28.0 (IBM, NY, USA). Descriptive statistics were presented as counts and percentages for categorical variables. For continuous variables, means and standard deviations were reported if the data distribution was normal; otherwise, medians and minimum-maximum values were used. Student’s *t*-test or Mann–Whitney *U* test was employed to compare continuous variables between the two groups, depending on normality. Categorical variables were compared using the chi-square test. Receiver operating characteristic (ROC) curve analysis was employed to determine the optimal cutoff values for the neutrophil-to-ferritin ratio (NFR) and SII, with the presence of hematological etiology as the outcome of interest. Binary logistic regression analysis with the enter method was employed for multivariate analysis, including factors with a p-value below 0.2 in univariate analysis. A p-value of less than 0.05 was considered significant for statistical significance in multivariate analysis.

## Results

### Patients characteristic

The demographic characteristics and laboratory parameters of the hospitalized patients are outlined in Table 1. Among the patients, 54% were female, with a median age of 52. The predominant comorbidities observed were hypertension 27% and diabetes mellitus 19%. Regarding the causes of FUO, 26 patients were found to have hematologic etiologies, and 74 patients had nonhematologic etiologies. The leading nonhematologic diagnoses were infections 43% and rheumatologic pathologies 24%. The median duration from symptom onset to diagnosis was 7 weeks.

**Table 1:** General patient characteristics (N: 100).

Parameters	*n*	
Age (median)	52	(19–82)
Sex, female (%)	46	46
Comorbidities		
Diabetes mellitus, (%)	19	19
Hypertension, (%)	27	27
Coronary artery disease, (%)	6	6
Chronic renal failure, (%)	7	7
Diagnosis subgroups		
Hematologic, (%)	26	26
Infection, (%)	43	43
Rheumatologic, (%)	24	24
Solid organ malignancy, (%)	5	5
Sarcoidosis, (%)	1	1
Subacute thyroiditis, (%)	1	1
Time to diagnosis, week (median)	7	(4–45)
Hemoglobin, g/dl (mean ± standart deviation)	10.2	± 1.6
Neutrophil, 10^9^/L (median)	5.3	(0.01–27.3)
Lymphocyte, 10^9^/L (median)	1.4	(0.2–8.5)
Monocyte, 10^9^/L (median)	0.6	(0.01–31.6)
Platelets, 10^9^/L (mean±standart deviation)	259.5	± 139
Ferritin, µg/L (median)	573	(7–40.000)
CRP, mg/L (mean ± standart deviation)	107.4	± 64.4
ESR, mm/h (median)	54	(8-120)
NLR, (median)	4.2	(0.01–32.3)
PLR, (median)	181.5	(10-1150)
SII, (median)	941.3	(0.8–7016)
SIRI, (median)	2.95	(0.1–45.9)
NFR, (median)	10.6	(0.01–830)

*CRP* C-reactive protein, *ESR* erythrocyte sedimentation rate, *NLR* neutrophil lymphocyte ratio, *PLR* platelet lymphocyte ratio, *SII* serum inflammation index, *SIRI* systemic inflammation response index, *NFR* neutrophil ferritin ratio.

**Table 2:** Distribution of reasons for FUO (n: 100).

Diagnoses	*n*	%
**Hematologic**	26	
Non-Hodgkin lymphoma	14	53.9
Hodgkin’s lymphoma	8	30.8
Acute myeloid leukemia	3	11.5
Acute lymphoblastic leukemia	1	3.8
**Non-hematologic**	74	
Still’s disease	10	13.5
Vasculitis	8	10.8
SLE	3	4.1
Autoinflammatory disease	3	4.1
Nonspecific infection	23	31.1
Infective endocarditis	7	9.4
Tuberculosis	7	9.4
*Brusella*	3	4.1
Q fever	3	4.1
Metastatic adenocancer	3	4.1
RCC	2	2.7
Sarcoidosis	1	1.3
Subacute Tthyroiditis	1	1.3

*SLE* systemic lupus erythematosus, *RCC* renal cell carcinoma.

Table 2 illustrates the distribution of causes of FUO among the patients. Lymphomas were the predominant disease in patients with hematologic etiology, accounting for 84.7% of cases. Among lymphomas, non-Hodgkin’s lymphoma constitutes 53.9% of cases, while Hodgkin’s lymphoma was present in 30.8% of cases. Nonspecific infections were the most common cause among nonhematologic etiologies, and Still’s disease emerged as the primary etiology among rheumatologic diseases.

### Univariate and multivariable analyses for predicting FUO etiology

The comparative analysis presented in Table 3 outlines the differences between patients with hematologic and nonhematologic diagnoses. The data shows no significant variations in age and gender distribution between the two groups. However, the median time from symptom onset to diagnosis was notably longer for the hematologic group (8 weeks) compared to the nonhematologic group (*p* = 0.024). Additionally, patients with hematologic diagnoses exhibited significantly lower neutrophil, lymphocyte, and platelet counts upon hospitalization, as well as significantly elevated ferritin levels (*p* = 0.003). While inflammation scores such as C-reactive protein (CRP), erythrocyte sedimentation rate (ESR), NLR, PLR, and SIRI were comparable between the two groups, SII levels were notably lower (median of 651.5) in patients with hematologic diagnoses, with this difference being statistically significant (*p* = 0.015).

**Table 3:** Evaluation of patient characteristics according to diagnosis subgroups.

Parameters	Hematologic diagnosis *n*	Non-hematologic diagnosis *n*	*p*-value
Age (median)	57 (19–72)	51(19–82)	0.82^a^
Gender, female (%)	8 (30.8)	38 (51.3)	0.11^b^
Time to diagnosis, week (median)	8 (4–45)	7 (4–16)	**0.024** ^**a**^
Hemoglobin, g/dl (mean ± SD)	10.02 ± 1.8	10.26 ± 1.55	0.52^a^
Neutrophil, 10^9^/L (median)	3.5 (0.01–20.5)	6.2 (0.01–27.3)	**< 0.001** ^**a**^
Lymphocyte, 10^9^/L (median)	1.05 (0.2–8.5)	1.5 (0.5–3.4)	**0.002** ^**a**^
Monocyte, 10^9^/L (median)	0.65 (0.11–31.6)	0.63 (0.01–10.4)	0.89^a^
Platelets, 10^9^/L (mean ± SD)	185.5 ± 125	285.4 ± 134	**0.001** ^**c**^
Ferritin, µg/L (median)	1040.5 (141 − 40.000)	468.5 (7–40.000)	**0.003** ^**a**^
CRP, mg/L (mean ± SD)	118.8 ± 68.8	98.07 ± 59.5	0.11^c^
ESR, mm/h (median)	55 (8–120)	53 (13–120)	0.89^a^
NLR (median)	4.05 (0.01–32.3)	4.4 (0.1–19.9)	0.36^a^
PLR (median)	173.4 (10–1150)	181.5 (18.1–496)	0.90^a^
SII (median)	651.5 (1.1–3483)	1139.9 (0.8–7016)	**0.015** ^**a**^
SIRI (median)	2.9 (0.1–19.4)	3.05 (0.1–45.9)	0.61^a^
NFR (median)	2.11 (0.01–31.6)	14.72 (0.01–830)	**< 0.001** ^**a**^

Significant values are given in bold.

*SD* standart deviation, *CRP* C-reactive protein, *ESR* erythrocyte sedimentation rate, *NLR* neutrophil lymphocyte ratio, *PLR* platelet lymphocyte ratio, *SII* serum inflammation index, *SIRI* systemic inflammation response index, *NFR* neutrophil ferritin ratio.

^a^Mann–Whitney *U*.

^b^Continuity correction.

^c^Independent sample *t* test.

**Table 4:** Univariate and multivariate logistic regression analysis for predicting hematologic diagnosis in cases of fever of unknown cause.

Factor	Univariate Analysis				Multivariate Analysis			
	OR	95% Cl		p-value	OR	95% Cl		p-value
		Lower	Upper			Lower	Upper	
Age (years)	0.997	0.972	1.023	0.808				
Gender (male [RC] vs. female)	2.375	0.919	6.137	0.074	2.496	0.821	7.587	0.107
Hemoglobin, g/dl	1.096	0.831	1.447	0.516				
Neutrophil, 10^9^/L	1.178	1.035	1.341	0.013	1.158	0.990	1.355	0.067
Lymphocyte, 10^9^/L	1.533	0.844	2.783	0.161	0.787	0.439	1.413	0.423
Monocyte, 10^9^/L	0.925	0.803	1.066	0.280				
Platelets, 10^9^/L	1.006	1.002	1.010	0.003	1.005	1.000	1.011	0.072
Ferritin, µg/L	1.000	1.000	1.000	0.195	1.000	1.000	1.000	0.756
CRP, mg/L	0.997	0.990	1.004	0.349				
ESR, mm/h	1.007	0.990	1.025	0.402				
NLR	0.992	0.901	1.092	0.873				
PLR	0.998	0.995	1.001	0.262				
SIRI	1.022	0.941	1.109	0.607				
SII (low [RC] vs. high)	2.250	0.871	5.814	0.094	0.333	0.061	1.822	0.205
NFR (low [RC] vs. high)	6.533	2.329	18.331	< 0.001	4.525	1.380	14.843	**0.013**

Significant values are given in bold.

*OR* odds ratio, *CI* confidential interval, *RC* reference category, *Hb* hemoglobin, *Plt* platelets, *CRP* C-reactive protein, *ESR* erythrocyte sedimentation rate, *NLR* neutrophil lymphocyte ratio, *PLR* platelet lymphocyte ratio, *SII* serum inflammation index, *SIRI* systemic inflammation response index, *NFR* neutrophil ferritin ratio.

**Fig. 2:**
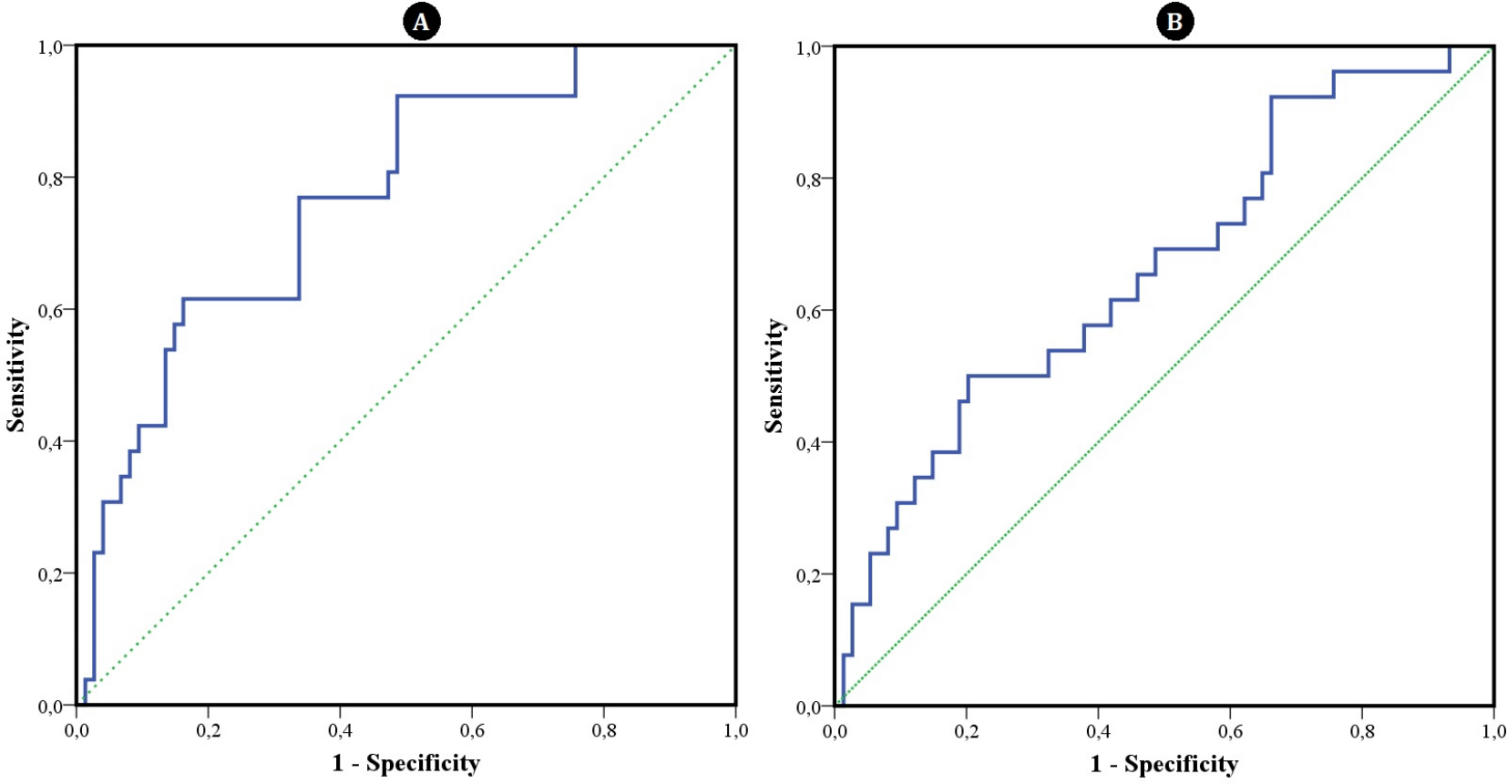
(**A**) ROC Analysis of the NFR for predicting hematologic etiology, (**B**) ROC Analysis of the SII for predictinghematologic etiology.

The median NFR was 2.11 in the hematologic group and 14.72 in the nonhematologic group, showing a statistically significant difference (*p* < 0.001). In the univariate analysis, both SII and NFR emerged as significant markers for predicting hematologic etiology in FUO patients. Figure 2 illustrates the ROC curve analysis of SII and NFR, which was conducted to establish a cutoff value predictive of hematologic diagnosis. The ROC Curve analysis for SII revealed a cutoff value of < 1139 (AUC: 0.66, sensitivity: 69.2%, specificity: 50%, *p* = 0.01), and the ROC Curve analysis for NFR identified a cutoff value of < 8.53 (AUC: 0.77, sensitivity: 76.9%, specificity: 66.2%, *p* < 0.001). The results of logistic regression analysis are presented in Table-4. Upon multivariate analysis, NFR (OR 4.525; 95% CI 1.380-14.483; *p* = 0.013) emerged as an independent predictor of hematologic etiology in FUO patients. Gender, neutrophil, lymphocyte, platelet, ferritin levels, and SII did not reach statistical significance in the multivariate analysis.

## Discussion

Identifying the underlying cause of FUO is crucial as it guides both the treatment approach and patient survival. The three primary causes of FUO are infections, malignancies, and rheumatologic diseases, with infectious causes accounting for 38–45% of cases, malignancies for 15–20%, and rheumatologic diseases for 10–15%^19^. The wide range of potential etiologies and the need for invasive and non-invasive diagnostic procedures make diagnosing FUO challenging. Delayed diagnosis is particularly common in cases where hematologic malignancies are responsible for FUO, leading to low overall survival rates^17^. To our knowledge, the present study is the first to evaluate inflammation scores as predictors of hematologic etiology in FUO patients. We found that the NFR is an independent predictor of hematologic etiology, meaning that NFR can be valuable for identifying hematological causes of FUO.

It can be challenging to determine the cause of fever in patients with hematologic malignancies, especially in those who have recently been diagnosed with leukaemia or lymphoma^20^. Primary disease-associated fever and the development of hemophagocytic lymphohistiocytosis may become more prominent once infectious causes have been ruled out^21^. In the group where chemotherapy was initiated after diagnosis, patients mainly present with infection-related febrile neutropenia^22^. In our study, all patients in the hematologic group were newly diagnosed and did not have any concomitant infectious pathologies.

In the current literature, numerous studies have investigated the parameters associated with predicting the causes of FUO. The majority of these studies have primarily focused on infectious agents and rheumatologic diseases^23–26^. Most of the research pertaining to predicting hematologic causes in these patients has emphasized the diagnostic accuracy of bone marrow biopsy^12,27^. For instance, in a study by Wang et al. involving 85 FUO patients, the study explored parameters that predict the diagnostic outcome of bone marrow biopsy. The developed bone marrow score incorporated neutropenia and elevated ferritin, with ferritin elevation being identified as the most influential laboratory parameter^27^, supporting our findings that ferritin elevation and neutropenia are significant predictors of hematologic causes, and the NFR may provide predictive value in identifying such etiologies.

In cases of infectious FUO, moderate ferritin elevation is typically accompanied by neutrophilic leukocytosis. Notably, when FUO patients exhibit ferritin levels exceeding 500 ng/ml upon hospitalization, non-infectious diagnoses tend to be more prevalent as the etiologic cause^28^. For example, in a study by Kim et al., the mean ferritin level was 282.4 ng/ml in patients with infectious FUO. In comparison, it was notably higher at 1818.2 ng/ml in patients diagnosed with hematologic malignancy^29^. Similarly, our study revealed significantly higher ferritin levels in FUO patients with hematologic malignancies (1040.5 µg/L) compared to a moderate increase observed in the nonhematologic group (468.5 µg/L).

Ferritin elevation is also observed in chronic inflammation conditions other than infection and malignancy. Especially in autoimmune diseases such as rheumatoid arthritis, adult-onset Still’s disease (AOSD), and malignancies, increased hepcidin restricts the mobilization of iron in the body and causes it to be stored in macrophages, and serum iron level decreases. As a result of both the decrease in serum iron level and the acute phase reaction, a significant increase in serum ferritin level occurs^30–32^. In hematologic malignancies, ferritin levels are markedly increased, especially as a result of cytokines secreted by tumor-associated macrophages^33^.

Although a high ferritin level indicates non-infectious FUO cases, it is insufficient for differential diagnosis between rheumatologic diseases and malignancies. Among non-infectious etiologies, approximately 15–20% of non-infectious cases are caused by AOSD^34,35^ and approximately 60% of malignant etiologies are caused by lymphomas^19^ in different FUO series. Therefore, these two main groups are essential for differential diagnosis after infectious-noninfectious differentiation. The Yamaguchi criteria are frequently used in diagnosing AOSD; one of the major criteria is neutrophilic leukocytosis^16^. In studies on predicting AOSD cases in FUO patients, apart from clinical criteria such as the presence of arthralgia and sore throat, ferritin elevation, and neutrophilic leukocytosis were found to be parameters with high predictive value for the diagnosis of AOSD^24^. Similarly, the AF score developed by Ying et al. for diagnosing AOSD includes elevated neutrophils and ferritin^23^. In contrast to rheumatologic diseases, leukopenia and neutropenia are prominent in hematologic malignancies detected in FUO cases. In these cases, neutropenia develops due to factors such as bone marrow infiltration, prolonged time to diagnosis, and bone marrow suppression due to cytokine discharge^12,36^. In a study by Naito et al., leukopenia (< 4000 µg/L) in FUO patients was highly associated with hematologic malignancy^37^. Similarly, in a study evaluating the characteristics of lymphoma patients presenting with FUO clinic, neutropenia was found to be higher than in lymphoma patients who did not meet FUO criteria^36^. Wan et al. identified neutropenia as a significant parameter in the PET-based scoring system developed for differentiating between AOSD and lymphoma patients^38^. In our study, the neutrophil count was lower in the hematologic group (3500 µg/L vs. 6200 µg/L). When this low neutrophil count was evaluated together with high ferritin levels, the diagnostic power of the NFR ratio in predicting hematologic etiology increased.

Neutropenia, thrombocytopenia, lymphopenia, and anemia are common occurrences resulting from bone marrow suppression due to bone marrow infiltration or cytokine discharge in patients with hematologic malignancies^12,36^. Inflammation scores like NLR, PLR, SII, and SIRI, which are calculated using these parameters, are now frequently utilized for prognosis in both hematologic and oncologic malignancies^39,40^. Although the parameters included in these scores are often used in FUO cases, there are insufficient studies in the literature examining the impact of inflammation scores on differential diagnosis. Our research observed that neutrophil, lymphocyte, and platelet levels were lower in the group with hematologic etiology compared to the other patients, and the mean hemoglobin level was lower, although it did not reach statistical significance. Of the inflammation scores, only SII was found to be significant in univariate analysis for predicting hematologic etiology, but it lost its significance against NFR in multivariate analysis. Studies in the literature that emphasize hematological etiology include neutropenia and ferritin elevation and the presence of anemia and thrombocytopenia, but their diagnostic value is low^12,27,36^.

The limitations of our study include its retrospective design, being a single-center study, and not including laboratory parameters that may contribute to differential diagnosis, such as glycosylated ferritin and procalcitonin. The fact that geographical and sociocultural characteristics influence etiologic causes in FUO cases is among the limitations of our single-center study.

## Conclusions

Based on our study findings, the NFR ratio emerges as a valuable parameter for predicting hematologic causes in FUO cases, which are challenging to diagnose and can be life-threatening due to delayed diagnosis. A cutoff NFR value of < 8.53 enhances its predictive value. Further prospective studies involving larger patient cohorts are needed to validate our results.

## Data Availability

The datasets used and/or analyzed during the current study are available from the corresponding author upon reasonable request.
